# The revised international autoimmune hepatitis score in chronic liver diseases including autoimmune hepatitis/overlap syndromes and autoimmune hepatitis with concurrent other liver disorders

**DOI:** 10.1186/1740-2557-4-3

**Published:** 2007-06-29

**Authors:** Panagiotis A Papamichalis, Kalliopi Zachou, George K Koukoulis, Aikaterini Veloni, Efthimia G Karacosta, Lampros Kypri, Ioannis Mamaloudis, Stella Gabeta, Eirini I Rigopoulou, Ansgar W Lohse, George N Dalekos

**Affiliations:** 1 Dept of Medicine, Research Laboratory of Internal Medicine, Medical School, University of Thessaly, 22 Papakiriazi str, Larissa 41222, Greece; 2 Dept. of Medicine, Academic Liver Unit, Medical School, University of Thessaly, 22 Papakiriazi str, Larissa 41222, Greece; 3 Dept. of Pathology, Medical School, University of Thessaly, 22 Papakiriazi str, Larissa 41222, Greece; 4 Dept. of Medicine I, University Medical Center Hamburg-Eppendorf, Hamburg, Germany

## Abstract

**Background:**

We conducted a study in order to determine the usefulness and diagnostic value of International Autoimmune Hepatitis Group (IAHG) score in non-autoimmune hepatitis (AIH) hepatic disorders as well as in AIH/overlap syndromes and in cases with coexistence of AIH and other liver diseases.

**Methods:**

We applied the IAHG score in 423 patients with liver diseases excluding patients with AIH, AIH/overlap syndromes and AIH with concurrent other liver disease namely, patients with chronic hepatitis B (n = 109), chronic hepatitis C (n = 95), chronic hepatitis D (n = 4), alchoholic liver disease (n = 28), non-alcoholic fatty liver disease (n = 55), autoimmune cholestatic liver diseases (n = 77), liver disorders of undefined origin (n = 32) and with miscellaneous hepatic disorders (n = 23). 24 patients with AIH associated with any kind of liver disorder including 10 patients with AIH/overlap syndromes and 14 AIH with concurrent other liver disease were also investigated. 43 patients with AIH consisted the control group.

**Results:**

The specificity of the score was 98.1% while the sensitivity in unmasking AIH in patients with either AIH/overlap syndromes or AIH with concurrent other liver diseases was only 50% and 78.6%. In the binary logistic regression model, the presence of other autoimmune diseases (p < 0.001), the total histological score (p < 0.001) and positivity for autoantibodies (p < 0.05) were identified as independent predictors for the presnce of AIH/ovea syndromes o AI with concurren other liver diseass.

**Conclusion:**

The IAHG scoring system has very good specificity for excluding AIH in patients with chronic liver diseases but not that sensitivity in order to unmask AIH/overlap syndromes or AIH with concurrent other liver diseases. The presence of other autoimmune diseases or autoantibody markers in the absence of hepatitis viral markers should alarm physicians for the possible presence of AIH either as "pure" AIH or in association with other liver disorders (AIH/overlap syndromes or AIH with concurrent other liver diseases). Under these conditions, liver histology seems essential and it must always be included in the work up of hepatic patients.

## Background

Autoimmune hepatitis (AIH) is a chronic liver disease of unknown aetiology, characterized by female predominance, hypergammaglobulinemia, circulating autoantibodies, association with human leukocyte antigens (HLA) DR3 or DR4 and a favourable response to immunosuppression [1-4]. The diagnosis of AIH is based on the revised descriptive criteria for diagnosis of AIH reported by the International Autoimmune Hepatitis Group (IAHG) in 1999 [5].

However, some of the features that may support AIH diagnosis, such as elevated serum IgG, the detection of autoantibodies and histologically evident interface hepatitis, can occur with variable frequency in a wide range of other liver disorders [6-9]. The problem arising in diagnosis makes the decision for clinical management difficult as corticosteroids, which constitute the treatment of choice in AIH are usually contraindicated in these disorders as they can induce exacerbation of liver disease in cases of chronic viral hepatitis (CVH) or may deteriorate the steatosis in cases of non alcoholic fatty liver disease (NAFLD). Furthermore, the diagnosis and consequently the management is even more complicated in the presence of AIH/primary biliary cirrhosis (PBC) or AIH/primary sclerosing cholangitis (PSC) overlap syndromes as well as in cases with coincidence of AIH and other liver diseases (CVH, NAFLD, alcohol consumption, etc).

In an attempt to provide a template for diagnosing AIH in a quantitative and objective manner the IAHG codified in 1993 and updated in 1999 a scoring system for the diagnosis of definite or probable AIH [5,10]. The AIH score constitutes an objective way for assessing different groups of patients uniformly by including ten basic parameters as follows: the gender, the alkaline phosphatase (ALP) to alanino-aminotransferase (ALT) ratio, the serum globulins or IgG increase, the presence or absence of antinuclear antibodies (ANA), smooth muscle antibodies (SMA) or liver kidney micosomal antibodies type 1 (anti-LKM-1), the presence or absence of antimitochondrial antibodies (AMA), the presence or absence of hepatitis viral markers, the history of illicit drug use or alcohol abuse, the histological findings compatible or not with AIH and the presence of other autoimmune diseases [5].

The score is mainly used for research purposes in order to include homogenous population in research projects but occasionally it can be used also in the clinical practice particularly for the diagnosis of undetermined chronic liver diseases. Whether the application of the AIH scoring system is useful in cases of AIH/overlap syndromes and furthermore, in cases of AIH with concurrent other liver disease has not been extensively validated so far. Accordingly, we conducted a study in order to evaluate in a large cohort of patients with chronic liver diseases the diagnostic value of AIH score in other than AIH hepatic disorders, in AIH/PBC or AIH/PSC overlap syndromes as well as in coincidental cases of AIH with other liver diseases, as the correct diagnosis of such patients is crucial for their proper management. In addition, we analysed and compared patients' characteristics of each disease group in an attempt to find out autoimmune features in patients with liver diseases of non-autoimmune origin that may hamper the correct diagnosis and investigated the factors that may distinguish patients with AIH/overlap syndromes or with AIH accompanied by other liver diseases from patients with diverse non-AIH chronic liver diseases.

## Methods

### Patients

The medical records of 490 patients with diverse liver diseases for whom complete data for the calculation of the revised IAHG score were available, were reviewed retrospectively with the application of the IAHG scoring system. Data were collected at the time of the first admission of each patient in our department and/or visit to the outpatient clinic. For patients with more than one biopsy, the first one was taken into account. All patients in the study were evaluated in a uniform fashion and diagnosis was assessed according to conventional criteria, by two independent experienced investigators (G.N.D. and K.Z.). The human research review committee of the University of Thessaly, Medical School approved the study protocol.

The diagnosis of chronic hepatitis C (HCV) was based on clinical, laboratory and histologic evaluation as we described previously [11-13]. Briefly, all HCV patients included in the study met the following criteria: a) serologic evidence of chronic HCV infection as determined by the detection of antibodies to HCV (anti-HCV) using a third-generation enzyme immunoassay at least twice within six months and b) active virus replication as defined by the detection of HCV RNA using a polymerase chain reaction. The diagnosis of chronic hepatitis B (HBV) and hepatitis D (HDV) was based on clinical, laboratory and histologic evaluation as described by us previously [13-16] and according to the recent EASL International Consensus Conference on Hepatitis B [17].

Patients with PBC met the following criteria: elevated cholestatic enzymes, liver histology with PBC lesions and positivity for AMA (positive titre ≥ 1:40) detected by indirect immunofluorescence (IIFL) on in-house rodent tissue substrates and confirmed by a competitive enzyme linked immunosorbent assay (ELISA) with reference antibody directed against the E2 subunit of pyruvate dehydrogenase or of the branched chain keto acid dehydrogenase and by Western blotting using rat liver mitochondrial fractions following published protocols [18-22]. The diagnosis of PSC was based on biochemical or clinical signs of cholestasis, compatible liver histology, repeatedly AMA negativity by IIFL, Western blot or ELISA and/or typical findings on endoscopic retrograde cholangiopancreatography (ERCP) or magnetic resonance cholangiography (MRCP) [20,21]. The diagnosis of NAFLD was based on the presence of metabolic syndrome, exclusion of other causes of chronic liver disease (viral, autoimmune, drug and toxin induced) including alcohol abuse and compatible liver histology [23], while the presence of alcoholic liver disease (ALD) was documented on the grounds of history of increased alcohol consumption and compatible laboratory and histological lesions. Patients with liver disorders of undefined origin as well as with miscellaneous hepatic disorders were also included in the study. The first group of patients (n = 32) consisted of subjects with no confirmed diagnosis, in spite of a thorough clinical and laboratory investigation including liver biopsy and immunoserological assessment. The second group (miscellaneous hepatic disorders; n = 23) included patients with Wilson's disease (n = 3), secondary haemochromatosis (n = 5), Budd-Chiari syndrome (n = 2), drug-induced hepatitis (n = 5), benign liver tumors (n = 5) and one of each of the following conditions: a_1_-antithrypsin deficiency, benign cholestasis of pregnancy and Gilbert's syndrome (n = 3).

The diagnosis of AIH was based on the abovementioned revised descriptive criteria reported by the IAHG [5], while patients with AIH/overlap syndromes fulfilled the criteria for AIH diagnosis as well as those for the diagnosis of either PBC or PSC. Specifically, AIH/PBC and AIH/PSC overlap syndromes were defined by the coexistence of AIH and PBC or PSC diagnosed either simultaneously or consecutively according to previously described criteria [24-26]. Briefly, patients fulfilled at least two of the three criteria for each disease. AIH criteria: 1) positivity for autoantibodies (ANA, SMA or anti-LKM); 2) elevated serum IgG levels and 3) liver biopsy showing moderate or severe periportal or periseptal lymphocytic piecemeal necrosis; PBC criteria: 1) cholestatic biochemical profile 2) positivity for AMA and 3) histological lesions compatible with PBC; PSC criteria: 1) cholestatic biochemical profile, 2) specific cholangiographic features on ERCP or MRCP characteristic of PSC and 3) histological changes characteristic of PSC. The diagnosis of coincidental existence of AIH and CVH, NAFLD or ALD was based on the descriptive criteria for AIH diagnosis [5,10] along with those for CVH, NAFLD and ALD as mentioned above [9,20]. In particular, the diagnosis of cases with AIH and concurrent CVH was based on the criteria described by us previously [9,11-16,20]. In brief, all these cases had serologic and virologic evidence of HBV, HCV or HDV infections, while they had increased levels of aminotransferases with normal cholestatic enzymes, diffuse hypergammaglobulinaemia, detectable ANA, SMA or anti-LKM in high titres, seronegativity for AMA, absence of other aetiological factors such as, alcohol consumption or use of known hepatotoxic drug and a liver biopsy with characteristic lesions of AIH [5,10].

The 490 patients of the study were divided into three groups:

i). Patients with several liver diseases excluding AIH, AIH/overlap syndromes and coexistence of AIH with other liver diseases (n = 423). This group included 109 patients with chronic HBV infection, 95 with chronic HCV infection, 4 with chronic HDV infection, 28 with ALD, 55 with NAFLD, 51 with PBC, 26 with PSC (totally 77 patients with autoimmune cholestatic liver diseases; ACLD), 32 with liver disorders of undefined origin and 23 patients with miscellaneous hepatic disorders.

ii). Patients with AIH associated with any kind of liver disorder (n = 24) including 7 patients with AIH/PBC, 3 with AIH/PSC (totally 10 patients with AIH/overlap syndromes), 4 with AIH/chronic HBV infection, 3 with AIH/chronic HCV infection, 1 with AIH/chronic HDV infection, 4 with AIH/NAFLD and 2 patients with AIH/ALD (totally 14 patients with coincidence of AIH and other liver disease).

iii). Patients with AIH (n = 43), which constitute the disease control group.

Serum levels of ALT, g-glutamyl-transpeptidase (g-GT), ALP, IgG and γ-globulins were determined using standard techniques. ANA, SMA, anti-LKM and AMA were detected by IIFL on HEp-2 cells and rat liver-kidney-stomach cryostat sections using standard protocols and accepting titers ≥ 1:40 as positive in all cases [4,27-31]. For statistical reasons patients were divided into two groups [positive (pos)/negative (neg)] for each of the following parameters of the scoring system: ALP/ALT ratio (pos: score +2, neg: score 0 or -2), serum γ-globulins or IgG above the upper normal limit (pos: score ≥ 1, neg: score 0), detection of ANA, SMA or anti-LKM (pos: titre ≥ 1:40), total score from the evaluation of liver biopsy (pos: score ≥ 1, neg: score -11 to 0).

### Statistical analysis

Results are expressed as mean ± SD. Data were analysed by x^2 ^(two-by-two with Yate's correction), Fisher's exact test, Mann-Whitney U-test (MWU), Kruskal-Wallis test and Spearman's rank correlation (r), where applicable. The variables significant in the univariate analysis entered a binary logistic regression model. Two-sided p values less than 0.05 were considered statistically significant. Confidence intervals (95% CI) were determined using the formula P = p ± 1.96 (pq/n)^1/2 ^where p is the frequency, q is 1-p and n is the number of individuals tested from each group. The Documenta Geigy Scientific Table (Ciba-Geigy Ltd, Basle, Switzerland, 1972 7^th ^edition) was used for 95% CI when fewer than 41 individuals were tested.

## Results

The relevant demographic, clinical, biochemical, serological, and histological data required for the calculation of the revised IAHG score in the study population are shown in Table 1.

**Table 1:** Patients' characteristics.

*Parameters*	Patients with liver diseases* (n = 423)	Patients with coincidence of AIH and any kind of liver disorder (n = 24)**	*Patients with AIH/overlap syndromes (n = 10)*	*Patients with coincidence of AIH and other liver disease (n = 14)*	*Patients with AIH (n = 43)*
Gender (F/M)	231/192	12/12	5/5	7/7	33/10
Age mean ± SD (years)	51.6 ± 22.7	54.9 ± 18.6	49.6 ± 21.6	57.7 ± 15.5	52.3 ± 15.7
Serum globulin or IgG above normal (pos/neg; pos = score ≥ 1)	202/221	17/7	7/3	10/4	34/9
Alcohol abuse (>60 g/day: yes/no)	98/325	3/21	0/10	3/11	1/42
Drug use (yes/no)	25/398	0/24	0/10	0/14	1/42
Other autoimmune diseases (yes/no)	24/399	7/17	5/5	2/12	18/25
ANA or SMA or anti-LKM (pos/neg) (positive titre ≥ 1:40)	335/88	23/1	9/1	14/0	43/0
AMA (pos/neg)	49/374	4/20	4/6	0/14	0/43
Histology (pos/neg) (pos = score > 0, neg = score ≤ 0)	58/365	13/11	3/7	10/4	35/8
Total histologic score ± SD	-4 ± 3.3	0.38 ± 3.72	-2.1 ± 4.4	2.14 ± 1.7	2.73 ± 1.98
Median (range)	-5 (-11 to +5)	1 (-8 to +4)	0 (-8 to +4)	3 (0 to +4)	3 (-3 to +5)

Abbreviations are same as in text. *Excluding AIH patients (n = 43) and patients with coincidence of AIH and any kind of liver disorder (n = 24); **Means the coexistence of AIH with any kind of other liver disease; M = male; F = female.

### Specificity and sensitivity of IAHG score

The overall specificity (correctly negative/correctly negative + false positive) of the revised IAHG scoring system for the diagnosis of AIH in the context of the presence of another liver disease was 98.1% (95% CI: 96.8–99.4%) (Table 2). In more detail, the specificity of the revised IAHG scoring system for each liver disease was: 99.1% (95% CI: 97.3–100%) in HBV, 98.9% (95% CI: 97–100%) in HCV, 96.4% (95% CI: 91.5–100%) in NAFLD, 96.1% (95% CI: 90.7–100%) in ACLD namely 98% (95% CI: 93–100%) in PBC and 92.3% (95% CI: 82.1–100%) in PSC, 96.9% (95% CI: 90.9–100%) in patients with liver disorders of undefined origin and 100% in patients with ALD, HDV and miscellaneous hepatic disorders (Table 2).

**Table 2:** Specificity of the IAHG scoring system.

Patients with liver diseases* (n = 423)	*Specificity*
HBV (n = 109)	108/109 (99.1)
HCV (n = 95)	94/95 (98.9)
HDV (n = 4)	4/4 (100)
ALD (n = 28)	28/28 (100)
NAFLD (n = 55)	53/55 (96.4)
ACLD (n = 77)	74/77 (96.1)
PBC (n = 51)	50/51 (98)
PSC (n = 26)	24/26 (92.3)
Liver disorders of undefined origin (n = 32)	31/32 (96.9)
Miscellaneous hepatic disorders (n = 23)	23/23 (100)
	Overall specificity: 415/423 (98.1%)

Abbreviations are same as in text. *Excluding AIH patients (n = 43) and patients with coincidence of AIH and any kind of liver disorder (n = 24).

Only 8 out of 423 patients had an AIH score between 10 and 15 (probable AIH), while none of these patients had a score above 15 (definite AIH). During a follow-up period of 30–52 months, 7 patients did not develop other features supportive of AIH diagnosis, while they responded favourably to the treatment given according to their original diagnosis. The eighth patient that was classified initially as liver disorder of undefined origin has been lost in follow-up.

The sensitivity (correctly positive/correctly positive + false negative) of the revised IAHG scoring system for detecting AIH in association with any kind of other liver disease (n = 24) was 66.7% (95% CI: 44.7–84.4 %) with 8 out of 24 patients (3 with AIH/PBC, 2 with AIH/PSC, 1 with AIH/ALD, 1 with AIH/HBV and 1 with AIH/HCV) achieving aggregate score <10. The remaining 16 patients (4 with AIH/PBC, 1 with AIH/PSC, 1 with AIH/ALD, 4 with AIH/NAFLD, 3 with AIH/HBV, 1 with AIH/HDV and 2 with AIH/HCV) had an AIH score between 10 and 15. In more detail, the sensitivity of the score for detecting AIH in patients with AIH/PBC or AIH/PSC overlap syndromes (n = 10) was 50% (95% CI: 19–81%) with 5 out of 10 patients with AIH/overlap syndromes achieving score <10. However, the score was more sensitive in the detection of the coincidence of AIH and other liver disease (n = 14): 78.6% (95% CI: 57.7–100%) with only 3 out of 14 patients achieving score <10.

### Parameters that associated with increased AIH score in patients with chronic liver diseases (n = 423)

In the univariate analysis, parameters that significantly differed between patients with liver diseases achieving AIH score between 10 and 15 (n = 8) and those having a negative score (aggregate score less than 10; n = 415) were as follows: a) positive (≥1) score in liver biopsy (p = 0.015) and b) total score from the biopsy (p < 0.001) (Table 3). After binary logistic regression analysis we found that the total histological score obtained from liver biopsy, was the only independent factor that significantly associated (p = 0.003) with a probable AIH score in non-AIH patients with chronic liver diseases. Finally, the aggregate score was significantly higher in the group of patients with probable score compared to those achieving a negative AIH score (11.63 ± 1.19 vs 2.29 ± 4.07; p < 0.001; Table 3).

**Table 3:** Comparisons of the parameters of the IAHG scoring system between patients with chronic liver diseases* achieving a probable (n = 8) and a negative AIH score (n = 415).

*Characteristics*	Negative score no. of patients (%)	Probable score no. of patients (%)	P-value
Gender			
Female	225 (54.2)	6 (75)	NS
Male	190 (45.8)	2 (25)	
ALP/ALT ratio			
Positive: score +2	330 (79.5)	8(100)	NS
Negative: score 0, -2	85 (20.5)	0	
Serum globulin or IgG above normal			
Positive (score ≥ 1)	196 (47.2)	6 (75)	NS
Negative (score 0)	505 (57.9)	2 (25)	
ANA, SMA or anti-LKM			
Positive (titre ≥ 1:40)	327 (78.8)	8 (100)	NS
Negative	88 (21.2)	0	
AMA			
Positive (titre ≥ 1:40)	48 (11.6)	1 (12.5)	NS
Negative	367 (88.4)	7 (87.5)	
Hepatitis viral markers			
Yes	214 (51.6)	2 (25)	NS
No	201 (48.4)	6 (75)	
History of illicit drug use			
Yes	25 (6)	0	NS
No	390 (94)	8 (100)	
Average alcohol intake			
<25 g/day	318 (76.6)	7 (87.5)	NS
>60 g/day	97 (23.4)	1 (12.5)	
Histological score			
Positive (score > 0)	54 (13)	4 (50)	0.015**
Negative (score ≤ 0)	361 (87)	4 (50)	
Aggregate histological score (mean ± SD)	-4.13 ± 3.3	0.88 ± 2.8	<0.001***
Other autoimmune disease			
Yes	23 (5.5)	1 (12.5)	NS
No	392 (94.5)	7 (87.5)	
Aggregate AIH score (mean ± SD)	2.29 ± 4.07	11.63 ± 1.19	<0.001***

Abbreviations are same as in text; NS: not statistically significant; SD: standard deviation; *Excluding AIH patients (n = 43) and patients with coincidence of AIH and any kind of liver disorder (n = 24); **Fisher's exact test; ***Mann-Whitney U test;

### Comparisons between groups

In total (n = 24), patients with AIH/overlap syndromes and AIH with concurrent other liver diseases had significantly more frequently a positive score (≥1) in liver biopsy (p < 0.001), higher total histological score (p < 0.001), higher frequency of a positive score for serum globulin or IgG increase (p = 0.035) and higher frequency of concurrent autoimmune diseases (p = 0.001) compared to those with liver diseases other than AIH (n = 423; Table 4). In addition, patients with AIH/overlap syndromes and AIH with concurrent other liver diseases (n = 24) tended to have more frequently autoantibodies detection compared to those (n = 423) with liver diseases other than AIH (p = 0.06; Table 4). However, when the variables, which were significant in the univariate analysis, entered the binary logistic regression model, the total histological score (p < 0.001), the seropositivity for autoantibodies (p < 0.05) and the presence of other autoimmune diseases (p < 0.001) were identified as independent predictors for the presence of AIH associated with any kind of other liver disorders.

**Table 4:** Comparisons of the parameters of the IAHG scoring system between patients with chronic liver diseases* (n = 423) and patients with coincidence of AIH and any kind of liver disorder (n = 24).

*Characteristics*	Patients with liver diseases* no. of patients (%)	Patients with coincidence of AIH and any kind of liver disorder no. of patients (%)	P-value
Gender			
Female	231 (54.6)	12 (50)	NS
Male	192 (45.5)	12 (50)	
ALP/ALT ratio			
Positive: score +2	338 (79.9)	20 (83.3)	NS
Negative: score 0, -2	85 (20.1)	4 (16.7)	
Serum globulin or IgG above normal			
Positive (score ≥ 1)	202 (47.8)	17 (70.8)	0.035**
Negative (score 0)	221 (52.2)	7 (29.2)	
ANA, SMA or anti-LKM			
Positive (titre ≥ 1:40)	335 (79.2)	23 (95.8)	0.06***
Negative	88 (20.8)	1 (4.2)	
AMA			
Positive (titre ≥ 1:40)	49 (11.6)	4 (16.7)	NS
Negative	374 (88.4)	20 (83.3)	
Hepatitis viral markers			
Yes	216 (51.1)	8 (33.3)	NS
No	207 (48.9)	16 (66.7)	
History of illicit drug use			
Yes	25 (5.9)	0	NS
No	398 (94.1)	24 (100)	
Average alcohol intake			
<25 g/day	325 (76.8)	21 (87.5)	NS
>60 g/day	98 (23.2)	3 (12.5)	
Histological score			
Positive (score > 0)	58 (13.7)	13 (54.2)	<0.001**
Negative (score ≤ 0)	365 (86.3)	11 (45.8)	
Aggregate histological score (mean ± SD)	-4 ± 3.3	0.38 ± 3.72	<0.001****
Other autoimmune disease			
Yes	24 (5.7)	7 (29.2)	0.001**
No	399 (94.3)	17 (70.8)	
Aggregate AIH score (mean ± SD)	2.5 ± 4.2	10 ± 4.1	<0.001****

Abbreviations are same as in text; NS: not statistically significant; SD: standard deviation; *Excluding AIH patients (n = 43) and patients with coincidence of AIH and any kind of liver disorder (n = 24); **Two by two x square after Yates' correction; ***Fisher's exact test; **** Mann-Whitney U test.

After comparisons of the parameters of IAHG score among the group of patients with chronic liver disorders other than AIH (n = 423), the group of patients with AIH/overlap syndromes (n = 10) and the group of patients with coexistence of AIH and other liver disease (n = 14) we found that (Table 5): (a) patients with AIH/overlap syndromes had significantly increased prevalence of AMA detection (p = 0.02), lower prevalence of viral hepatitis markers (p = 0.002), lower average alcohol intake (p = 0.07) and higher frequency of concurrent autoimmune diseases (p = 0.0005) compared to the group of patients with chronic liver disorders other than AIH; (b) patients with coincidence of AIH and other liver disease had significantly more frequently a positive score (≥1) in liver biopsy (p = 0.0005) and significantly higher total histological score (p = 0.0005) compared to the group of patients with chronic liver disorders other than AIH and (c) patients with AIH/overlap syndromes had significantly higher prevalence of AMA detection (p = 0.02), lower prevalence of viral hepatitis markers (p = 0.006) and lower total histological score (p = 0.01) compared to patients with AIH concurrent with other liver diseases. However, after binary logistic regression analysis the presence of other autoimmune disease was identified as the only independent predictor for the presence of AIH/overlap syndromes (p = 0.001) while the total histological score from liver biopsy was identified as the only independent predictor for the coexistence of AIH and other liver disease (p < 0.001).

**Table 5:** Comparisons of the parameters of the IAHG scoring system among patients with chronic liver diseases* (n = 423), AIH/overlap syndromes (n = 10) and patients with coincidence of AIH and other liver disease (n = 14).

Characteristics	Patients with liver diseases* no. of patients (%)	Patients with AIH/overlap syndromes no. of patients (%)	Patients with coincidence of AIH and other liver disease no. of patients (%)	P value
Gender				
Female	231 (54.6)	5 (50)	7 (50)	NS
Male	192 (45.4)	5 (50)	7 (50)	
ALP/ALT ratio				
Positive: score +2	338 (79.9)	8 (80)	12 (85.7)	NS
Negative: score 0, -2	85 (20.1)	2 (20)	2 (14.3)	
Serum globulin or IgG above normal				
Positive (score ≥ 1)	202 (47.8)	7 (70)	10 (71.4)	NS
Negative (score 0)	221 (52.2)	3 (30)	4 (28.6)	
ANA, SMA or anti-LKM				
Positive (titre ≥ 1:40)	335 (79.2)	9 (90)	14(100)	NS
Negative	88 (20.2)	1 (10)	0	
AMA				
Positive (titre ≥ 1:40)	49 (11.6)	4 (40)	0	0.009**
Negative	374 (88.4)	6 (60)	14(100)	
Hepatitis viral markers				
Yes	216 (51.1)	0	8 (57.1)	0.005**
No	207 (48.9)	10 (100)	6 (42.9)	
History of illicit drug use				
Yes	25 (5.9)	0	0	NS
No	398 (94.1)	10 (100)	14 (100)	
Average alcohol intake				
<25 g/day	325 (76.8)	10 (100)	11 (78.6)	0.013**
>60 g/day	98 (23.2)	0	3 (21.4)	
Histological score				
Positive (score > 0)	58 (13.7)	3 (30)	10 (71.4)	<0.001**
Negative (score ≤ 0)	365 (86.3)	7 (70)	4 (28.6)	
Aggregate histological score (mean ± SD)	-4± 3.3	-2.1± 4.4	2.14± 1.7	<0.001***
Other autoimmune disease				
Yes	24 (5.7)	5 (50)	2 (14.3)	<0.001**
No	399 (94.3)	5 (50)	12 (85.7)	
Aggregate AIH score (mean ± SD)	2.5 ± 4.23	9.3 ± 4.8	10.5 ± 3.7	<0.001***

Abbreviations are same as in text; NS: not statistically significant; SD: standard deviation; *Excluding AIH patients (n = 43) and patients with coincidence of AIH and any kind of liver disorder (n = 24); **Total x square (comparison amongst the three groups of patients; for statistical significance between groups see text of results; subheading *Comparison between groups*, second paragraph); ***Kruskal-Wallis test (comparison amongst the three groups of patients; for statistical significance between groups see text of results, subheading *Comparison between groups*, second paragraph).

Patients with AIH/overlap syndromes and coexistence of AIH with other liver diseases (n = 24) were significantly more frequent of male gender (50% vs 23.3%; p = 0.025), had higher frequency of a negative score (≤0) in liver biopsy (45.8% vs 18.6%; p < 0.02), higher frequency of AMA positivity (16.7% vs 0%; p < 0.02), higher prevalence of viral hepatitis markers (33.3% vs 0%; p < 0.001) and lower total histological score (0.38 ± 3.72 vs 2.73 ± 1.98; p = 0.004) compared with patients suffering from AIH (n = 43). However, the binary logistic regression analysis showed that male gender was the only independent factor that was able to differentiate the coexistence of AIH with any kind of liver disorder from the presence of isolated AIH (p = 0.025).

After comparisons of the parameters of IAHG scoring system among the group of patients with AIH (n = 43), the group of patients with AIH/overlap syndromes (n = 10) and the group of patients with coexistence of AIH and other liver disease (n = 14) we found that (Table 6): (a) patients with AIH/overlap syndromes had significantly increased prevalence of AMA detection (p = 0.001), more frequently a negative score (≤0) in liver biopsy (p = 0.003), and significantly lower total histological score (-2.1 ± 4.4 vs 2.73 ± 1.98, p = 0.001) compared to patients with AIH and (b) patients with coincidence of AIH and other liver disease had significantly higher prevalence of viral hepatitis markers (p < 0.001) and higher average alcohol intake (p < 0.05) compared to AIH patients. However, after binary logistic regression analysis the lower total histological score was the only independent predictive factor that was able to differentiate the presence of AIH/overlap syndromes from the presence of isolated AIH (p < 0.05), while the higher average alcohol intake was the only independent predictive factor that was able to differentiate the presence of AIH with concurrent other liver disease from the presence of isolated AIH (p = 0.02).

**Table 6:** Comparisons of the parameters of the IAHG scoring system among patients with AIH/overlap syndromes (n = 10), coincidence of AIH and other liver disease (n = 14) and AIH patients (n = 43).

Characteristics	Patients with AIH/overlap syndromes no. of patients (%)	Patients with coincidence of AIH and other liver disease no. of patients (%)	Patients with AIH no. of patients (%)	P value
Gender				
Female	5 (50)	7 (50)	33 (76.7)	NS
Male	5 (50)	7 (50)	10 (23.3)	
ALP/ALT ratio				
Positive: score +2	8 (80)	12 (85.7)	41 (95.3)	NS
Negative: score 0, -2	2 (20)	2 (14.3)	2 (4.7)	
Serum globulin or IgG above normal				
Positive (score ≥ 1)	7 (70)	10 (71.4)	34 (79.1)	NS
Negative (score 0)	3 (30)	4 (28.6)	9 (20.9)	
ANA, SMA or anti-LKM				
Positive (titre ≥ 1:40)	9 (90)	14 (100)	43(100)	0.055*
Negative	1 (10)	0	0	
AMA				
Positive (titre ≥ 1:40)	4 (40)	0	0	<0.001*
Negative	6 (60)	14(100)	43 (100)	
Hepatitis viral markers				
Yes	0	8 (57.1)	0	<0.001*
No	10 (100)	6 (42.9)	43 (100)	
History of illicit drug use				
Yes	0	0	1 (2.3)	NS
No	10 (100)	14 (100)	42 (97.7)	
Average alcohol intake				
<25 g/day	10 (100)	11 (78.6)	42 (97.7)	0.022*
>60 g/day	0	3 (21.4)	1 (2.3)	
Histological score				
Positive (score > 0)	3 (30)	10 (71.4)	35 (81.4)	0.005*
Negative (score ≤ 0)	7 (70)	4 (28.6)	8 (18.6)	
Aggregate histological score (mean ± SD)	-2.1 ± 4.4	2.14 ± 1.7	2.73 ± 1.98	0.001**
Other autoimmune disease				
Yes	5 (50)	2 (14.3)	25 (58.1)	NS
No	5 (50)	12 (85.7)	18 (41.9)	
Aggregate AIH score (mean ± SD)	9.3 ± 4.8	10.5 ± 3.7	17 ± 3.05	<0.001**

Abbreviations are same as in text; NS: not statistically significant; SD: standard deviation; * Total x square (comparison amongst the three groups of patients; for statistical significance between groups see text of results, subheading *Comparison between groups*, third paragraph); **Kruskal-Wallis test (comparison amongst the three groups of patients; for statistical significance between groups see text of results, subheading *Comparison between groups*, third paragraph).

**Figure 1:**
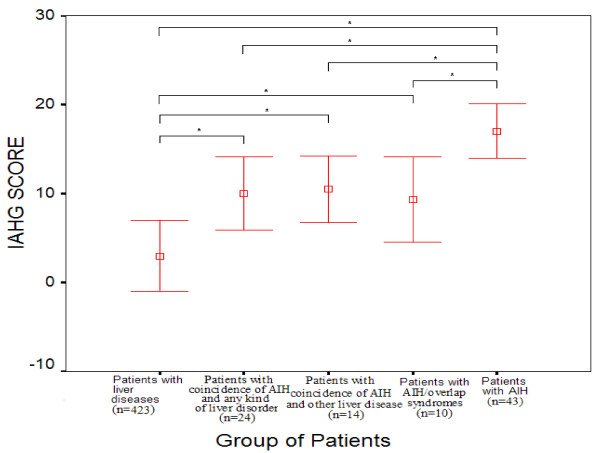
Mean ± SD revised IAHG score in each group of patients; *p < 0.001.

The aggregate IAHG score in the group of patients with coexistence of AIH and any kind of liver disease was significantly higher (10 ± 4.1; n = 24) compared to that found in patients with liver diseases after the exclusion of AIH patients, patients with AIH/overlap syndromes and patients with coincidence of AIH and other liver disease (2.5 ± 4.23; n = 423; p < 0.001; Table 4 and Figure 1) but significantly lower than the aggregate AIH score observed in patients with AIH (10 ± 4.1 vs 17 ± 3.05, respectively; p < 0.001, Figure 1). The aggregate IAHG score for each subgroup of patients with AIH/overlap syndromes (9.3 ± 4.8; n = 10) and patients with coincidence of AIH and other liver disease (10.5 ± 3.7; n = 14) was significantly higher compared to that found in patients with chronic liver diseases (2.5 ± 4.23; n = 423; p < 0.001; Figure 1) and significantly lower than the aggregate AIH score observed in patients with AIH (17 ± 3.05; n = 43; p < 0.001, Table 6 and Figure 1).

## Discussion

The major point arising from the present study is that the IAHG scoring system is highly specific for excluding AIH with specificity values ranging between 92.3%–100% but not so sensitive in order to detect the association of AIH with any kind of other liver disease. Indeed, the sensitivity of the score for the detection of either AIH/overlap syndromes or the coincidence of AIH with other liver disorders was rather low (50% and 78.6%, respectively). Furthermore, we observed that none of these patients with AIH/overlap syndromes or AIH with concurrent other liver disorders achieved a score above 15 (definite AIH), which is in accordance with a very recent report [32]. Therefore, our findings along with the assumption that the IAHG scoring system has been designed for study purposes only [5] may indicate the need for the development of a less complicated system for the diagnosis of AIH in every day use [33].

Among the large spectrum of liver diseases ALD, CVH and NAFLD are the most frequent entities that are being diagnosed in patients with liver disorders. However, their presence does not exclude by definition, the possibility of the coexistence of AIH. Under this context, non-organ specific autoantibodies commonly occur in patients with CVH [12,27,28,31,34-37], NAFLD [7,38,39] and ALD [40,41]. Furthermore, histological features similar to that observed in AIH as the presence of interface hepatitis may also occur in other liver diseases [8]. In this study, the specificity of the IAHG scoring system regarding the exclusion of AIH in patients with CVH, ALD and miscellaneous hepatic disorders was 98.9–100%. These findings demonstrate that the use of the IAHG scoring system provides a quite secure tool for the exclusion of AIH in patients with diverse liver diseases, which is in accordance with the findings of previous reports in patients with NAFLD, ALD and CVH [6,7,42].

In the group of ACLD we found that only 3/77 (3.9%) of patients achieved a probable AIH score. Previous studies reported the detection of probable or definite AIH score in 7.4% to 23% of PBC or PSC patients [24,43-47]. This discordance could be attributed to several demographic factors and differences regarding the genetic background. However, we believe that the main reason for the low incidence of a probable or definite AIH score among Greek PBC and PSC patients is rather due to the design of our study as in order to investigate the specificity of the score, we studied patients with PBC or PSC and features of AIH as a separate group (patients with AIH/overlap syndromes).

It is of interest that the aggregate AIH score was significantly lower in patients with AIH associated with any kind of liver disease (n = 24) compared to the respective score in AIH patients but significantly higher compared to that found in patients with chronic liver diseases (n = 423). The same findings observed when AIH/overlap syndromes and AIH concurrent with other liver diseases compared separately with AIH patients (n = 43) and patients with chronic liver diseases (n = 423), while the aggregate AIH score did not differ between patients with AIH/overlap syndromes and those with coexistence of AIH and other liver disease (Fig. 1). In addition, we showed that the total histological score, seropositivity for autoantibodies and the presence of other autoimmune diseases were independent predictive factors for the presence of AIH in association with any kind of other liver disorder. Separate analysis of the two groups showed that the presence of other autoimmune diseases and the total histological score were stable independent predictors for the identification of the presence of AIH/overlap syndromes and the coexistence of AIH and other liver disease, respectively. These findings are in accordance with previous reports [48] suggesting that the IAHG scoring system does provides some help for identifying the presence of AIH in association with any kind of liver diseases.

The presence of liver biopsy as an independent discriminative factor in almost all of our comparisons underlines its overall significance in the diagnosis of the AIH component of the individual case. For these reasons we believe that the second major point arising from our study is that the liver biopsy is essential in patients with liver diseases in whom there is evidence of autoimmunity and absence of evidence of HBV or HCV markers. Therefore, the close cooperation between clinicians and expertise hepatopathologists in an attempt to unmask the presence of AIH not only in the typical "pure" cases of AIH but also in difficult cases of the association of AIH with any kind of liver disorder seems mandatory.

On the other hand, the lower total histological score and the higher average alcohol intake were identified as independent predictors that were able to differentiate the presence of AIH/overlap syndromes and the presence of the coexistence of AIH and other liver disease from the presence of "pure" AIH, respectively. The latter findings underline the similarities of characteristics the two groups share in comparison with the "pure" cases of AIH making their discrimination quite difficult. At this point, the IAHG score works as an aggregation of all factors, and no factor by itself can contribute to the identification of each disease group (AIH, AIH/overlap syndromes and coexistence of AIH and other liver diseases).

## Conclusion

We demonstrated that the revised IAHG scoring system has very good specificity. On the contrary, the score is not very useful in detecting AIH/overlap syndromes or AIH with concurrent other liver diseases. Liver histology compatible with AIH, presence of other autoimmune diseases or autoantibody markers should alarm physicians for the possible presence of AIH either as "pure" AIH or as AIH with concurrent other liver disorders. Taken together, this study strongly suggests that liver biopsy must always be included in the work-up of hepatic patients with various indices of autoimmune manifestations and absence of evidence of hepatitis B or C, while the consultation with an expert liver pathologist is strongly recommended. However, as the number of our patients with overlap syndromes and those with coincident AIH is relatively small, our observations should be viewed with caution and need to be confirmed and tested in further multi-centre prospective studies with larger numbers of patients in order to define and establish appropriate diagnostic criteria for unmasking the coexistence of AIH with several other liver disorders including the AIH/overlap syndromes.

## Abbreviations

AIH, autoimmune hepatitis; HLA, human leukocyte antigens; IAHG, International Autoimmune Hepatitis Group; CVH, chronic viral hepatitis; NAFLD, non alcoholic fatty liver disease; PBC, primary biliary cirrhosis; PSC, primary sclerosing cholangitis; ALP, alkaline phosphatase; ALT, alanino-aminotransferase; ANA, antinuclear antibodies; SMA, smooth muscle antibody; anti-LKM-1, liver kidney microsomal type 1 antibody; AMA, antimitochondrial antibodies; HCV, hepatitis C virus; HBV, hepatitis B virus; HDV, hepatitis D; IIFL, indirect immunofluorescence; ELISA, enzyme linked immunosorbent assay; ERCP, endoscopic retrograde cholangiopancreatography; MRCP, magnetic resonance cholangiography; ALD, alcoholic liver disease; g-GT, g-glutamyl-transpeptidase; MWU, Mann-Whitney U-test.

## Competing interests

The author(s) declare that they have no competing interests.

## Authors' contributions

GND, KZ and AWL had the original idea for the study, wrote the study protocol, and along with EIR wrote the paper. GKK did the interpretation of liver biopsies. PAP, AV, EGK, LK and IM collected the whole data and along with EIR, SG and KZ did the statistical analysis and contributed to the final version of the paper. GND and AWL wrote the final version of the paper. All authors have seen and approved the final draft of the paper.
